# Effective improvement of the neuroprotective activity after spinal cord injury by synergistic effect of glucocorticoid with biodegradable amphipathic nanomicelles

**DOI:** 10.1080/10717544.2016.1256003

**Published:** 2017-02-06

**Authors:** YueLong Wang, Min Wu, Lei Gu, XiaoLing Li, Jun He, LiangXue Zhou, Aiping Tong, Juan Shi, HongYan Zhu, JianGuo Xu, Gang Guo

**Affiliations:** 1 State Key Laboratory of Biotherapy and Cancer Center and Department of Neurosurgery, West China Hospital, Sichuan University, and Collaborative Innovation Center for Biotherapy, Chengdu, PR China,; 2 Department of Radiology, Huaxi MR Research Center (HMRRC), West China Hospital, Sichuan University, Chengdu, PR China,; 3 National Laboratory of Medical Molecular Biology, Institute of Basic Medical Sciences, Chinese Academy of Medical Sciences and Peking Union Medical College, Beijing, PR China, and; 4 Laboratory of Stem Cell Biology, State Key Laboratory of Biotherapy, West China Hospital, Sichuan University, Chengdu, PR China

**Keywords:** Micelle, dexamethasone acetate, MPEG-PCL, spinal cord injury, synergistic effect

## Abstract

Dexamethasone acetate (DA) produces neuroprotective effects by inhibiting lipid peroxidation and inflammation by reducing cytokine release and expression. However, its clinical application is limited by its hydrophobicity, low biocompatibility and numerous side effects when using large dosage. Therefore, improving DA’s water solubility, biocompatibility and reducing its side effects are important goals that will improve its clinical utility. The objective of this study is to use a biodegradable polymer as the delivery vehicle for DA to achieve the synergism between inhibiting lipid peroxidation and inflammation effects of the hydrophobic-loaded drugs and the amphipathic delivery vehicle. We successfully prepared DA-loaded polymeric micelles (DA/MPEG-PCL micelles) with monodispersed and approximately 25 nm in diameter, and released DA over an extended period *in vitro*. Additionally, in the hemisection spinal cord injury (SCI) model, DA micelles were more effective in promoting hindlimb functional recover, reducing glial scar and cyst formation in injured site, decreasing neuron lose and promoting axon regeneration. Therefore, our data suggest that DA/MPEG-PCL micelles have the potential to be applied clinically in SCI therapy.

## Introduction

Spinal cord injury (SCI) is caused by motor vehicle accidents, recreation-related accidents, work-related accidents, falls and acts of violence, resulting in severe functional impairments, such as paraplegia and quadriplegia (Liu et al., 2012). As recently reported, regional incidence data for mainland China show 60.6 SCI per million population per year in Beijing and 23.7 in Tianjin province, meanwhile, the US show 721–4187 per million, with a median of approximately 853 per million (Lee et al., 2014). Disability due to SCI is a major global issue, affecting both young and elderly populations (Liu et al., 2012).

The pathological progression of SCI is often separated into two categories: primary injury and secondary injury (Shi et al., 2010; Snyder & Teng, 2012; Tyler et al., 2013). The secondary injury, followed by initiation of a number of secondary cellular and molecular responses, is mediated in part by vascular changes such as reperfusion and hemorrhage, breakdown of the blood spinal cord barrier (BCSB), electrolyte imbalances such as intracellular calcium overload, biochemical changes such as excitotoxicity due to neurotransmitter accumulation as in glutamate excitotoxicity, and various other stimuli such as lipid peroxidation, free radical production and edema (Hagg & Oudega, 2006; GhoshMitra et al., 2012; Wang et al., 2014; Saxena et al., 2015; Wert et al., 2016). Moreover, such second injury denotes the spread of damage from the original site to adjacent tissue through a cascade of deleterious reactions to the trauma (GhoshMitra et al., 2012; Snyder & Teng, 2012). Severely secondary injury, progressing rapidly after initial trauma and continuing for days or months, often results in recruitment of immune cells, apoptosis, disruption of synaptic connections and also axonal degradation, contraction and demyelination (Tyler et al., 2013).

Following surgical interventions that include early spinal decompression and stabilization surgery, the research for treating SCI can be broadly divided into two main areas: neuroprotection and regeneration (GhoshMitra et al., 2012; White-Schenk et al., 2015; Nazari-Robati et al., 2016). The goal of neural regeneration is to reestablish conduction in damaged spinal cords by promoting axonal regrowth. Neuroprotection focuses on preventing the spreading of secondary injury, reducing the subsequent damage (Hellal et al., 2011; Jörg Ruschel et al., 2015). Thus, early intervention to the secondary injury is very important. Currently, the drug used to treat the secondary injury during SCI process is an extremely large dose (30 mg/kg I.V. for the first hour, 5.4 mg/kg/h drip for 24 h) of methylprednisolone (MP) administering within the first 8 h post-injury (Bracken et al., 1997). This glucocorticoid drug is known as working through several mechanisms, including inhibition of lipid peroxidation and suppression of inflammation by reducing cytokine release and expression (Bracken et al., 1997; Xu et al., 1998). However, using high dose of glucocorticoid means serious side effects, such as myopathy, infections and gastric bleeding (Qian et al., 2005; Suberviola et al., 2008).

Micelles, formed from self-assembling amphiphilic molecules, consisting of a hydrophobic core and a hydrophilic shell, have been widely used in drug-delivery systems (DDSs) (Gao et al., 2015; Rezazadeh et al., 2016; Cheng et al., 2016; Li et al., 2016). Hydrophobic drug can be encapsulated in the core that could enhance drug’s circulation time (Xiong et al., 2016). Taking advantage of their size and flexibility, micelles are resistant to glomerular filtration, which could extend their retention time in blood (Gao et al., 2015). In recent years, polymeric micelles have drawn intense research interest in central nerve system disorder (Tyler et al., 2013; Saxena et al., 2015; Kabu et al., 2015). Some researches also reported that polymeric micelles could utilize for delivery of CNS drugs crossing the blood–brain barrier (BBB) (Gabathuler, 2010; Rezazadeh et al., 2016; Pardridge, 2016). Chen et al. improved the bioavailability of MP in the spinal cord using Poly(ethylene oxide)–poly(propylene oxide)–poly(ethylene oxide) (PEO–PPO–PEO, Pluronic) polymeric micelles as a delivery vehicle (Rezazadeh et al., 2016). And, they suspected that due to improved crossing of the BSCB or merely improved circulation time, the micelles were able to significantly improve bioavailability to the spinal cord.

It has been established that water-soluble polymers such as polyethylene glycol (PEG) and PEG-based nanoparticle (e.g. PEG–polyester, monomethoxy PEG–poly(D,L-lactic acid), PEG-silica, poly(lactic-co-glycolic acid)) could restore membrane integrity rapidly and effectively after injury in early stages of central nervous system (CNS) damage interfering with progressive secondary injury (Kim et al., 2009; Shi et al., 2010; Chen et al., 2013). These nanoparticles were proposed to act in CNS neuroprotection either by sealing the damaged axonal membranes, by an intrinsic antioxidant mechanism and also as drug-delivery system revealing some promising and satisfactory results. Dexamethasone acetate (DA) is a synthesized glucocorticoid, which work as the same as MP in human body. However, because of its poor solubility in water, the further application of DA has been restricted (Huang et al., 2016). Considering these drawbacks, a serial of micro/nanoparticle delivery-systems were developed to solve these problems (Park et al., 2012; Robinson et al., 2016; Janas et al., 2016). The objective of this study is to use a biodegradable polymer monomethyl poly(ethylene glycol)−poly(ɛ-caprolactone) (MPEG-PCL) as the delivery vehicle for DA to achieve the synergism between inhibiting lipid peroxidation and inflammation effects of the hydrophobic loaded drugs and sealing the damaged axonal membranes of the amphipathic delivery vehicle. We suppose the DA-loaded micelles (DA-M) could perform better neuroprotection effect than either DA or MPEG-PCL alone. In the following research, we successfully prepared DA-loaded polymeric micelles with monodispersed and characterized by particle size distribution, zeta potential, TEM, XRD and FT-IR, and the neuroprotection effect of DA micelles was investigated *in vivo*.

## Materials and methods

### Materials

Dexamethasone acetate (TCI, Shanghai, P.R. China), monomethyl poly(ethyleneglycol) (MPEG, Mn = 2000, Fluka, Darmstadt, Germany), ɛ-caprolactone (ɛ-CL, Mn = 2000, Alfa. Aesar, Shanghai, P.R. China), stannous octoate (Sn(Oct)_2_, (Sigma-Aldrich, Darmstadt, Germany) were used without purification. Methanol (HPLC grade) was purchased from Fisher Scientific (UK). Dichloromethane, acetone, dehydrated alcohol and paraformaldehyde were purchased from KeLong Chemicals (China). Other materials were identified as analytic reagent grade and used as received.

MPEG (2000)-PCL (2000) copolymer was synthesized according to our previous work (Qian et al., 2005). Briefly, MPEG and ɛ-CL were introduced into a dry glass ampoule under a nitrogen atmosphere. Sn(Oct)_2_ was then added into the reaction vessel under mild agitation, and the reaction system was kept at 130 °C for 6 h. The purified MPEG-PCL copolymer was kept in a desiccator before further use. The obtained copolymers were characterized by ^1^H NMR (molecular weight: 3900).

Sprague-Dawley rat (250 ± 20 g), purchased from the Beijing HFK bioscience CO., LED (Beijing, P.R. China), were provided with standard laboratory animal living conditions, including laboratory chow and tap water ad libitum controlled temperature between 20 and 22 °C, relative humidity of 50%−60%, and 12 h light − dark cycles. All animals were quarantined for 1 week before treatment. All animal procedures were performed following protocols approved by the Institutional Animal Care and Treatment Committee of Sichuan University (Chengdu, P.R. China). All rats were treated humanely throughout the experimental period.

### Preparation and characterization of DA-M

The DA micelles were prepared by a self-assembly method. DA and MPEG-PCL copolymer were codissolved in dichloromethane/acetone (v/v = 1:4), under mild stirring. Then, the mixed solution was evaporated using a rotary evaporator at 37 °C under gradually increasing vacuum degree, during which DA could be distributed in the MPEG-PCL copolymer, and they formed a homogeneous amorphous evaporation. Subsequently, the evaporation was dissolved in a certain volume (dependent on the designed DA concentration) of normal saline (NS) at 60 °C to self-assemble into DA-loaded micelles. DA-M was filtered through a 0.22 mm syringe filter (Millex-LG, Merck Millipore, Darmstadt, Germany), and was lyophilized and stored at 4 °C before use. Before lyophilization, the approach has been to avoid collisions by freezing the sample to 77 K in liquid nitrogen to give a rigid frozen matrix.

Particle size distribution and zeta potential of prepared DA-M were determined by dynamic light scattering (DLS) using a Malvern Nano-ZS 90 laser particle size analyzer at 25 °C. All results were the mean of three different samples, and all data were expressed as the mean ± SD.

The morphological characteristics of DA-M were examined by transmission electron microscope ((TEM, Tecnai G2 F20 S-TWIN electron microscope, FEI Co.). DA-M was diluted with distilled water and placed on a copper grid covered with nitrocellulose. Samples were negatively stained with phosphotungstic acid and dried at room temperature.

The prepared DA micelles were lyophilized into powder. Then, the powder was weighed and dissolved in methanol. Concentration of DA was determined by high-performance liquid chromatography (HPLC, Waters Alliance 2695) instrument and samples were diluted before measurement. The mobile phase contained 70% (v/v) methanol and 30% (v/v) ultrapure water, at a flow rate of 1 mL/min. Solvent-delivery system was equipped with a column heater and a plus autosampler. Detection was taken on a Waters 2996 detector. Chromatographic separations were performed on a reversed phase C18 column (4.6 × 250 mm-5 μm, Grace Analysis column), and the column temperature was kept at 28 °C.

Drug loading (DL) and encapsulation efficiency (EE) of DA-M were determined as follows. Briefly, 10 mg of lyophilized DA-M were dissolved in 0.1 mL of methanol. Concentration of DA in the solution was determined by HPLC. DL and EE of DA-M were calculated according to Equations (1) and (2):

(1) $$\mathrm{DL}\;=\;\frac{\underline{\mathrm{Drug}}}{\mathrm{Polymer}+\mathrm{Drug}}\times \;100\%$$

(2) $$\mathrm{EE}=\frac{\mathrm{Experimental}\;\mathrm{drug}\;\mathrm{loading}}{\mathrm{Theoretical}\;\mathrm{drug}\;\mathrm{loading}}\times 100\%$$

The crystallinity of the DA-M powder was performed by X-ray diffraction (XRD, X, Per Pro MPD DY 1291, PHILIPS, Amsterdam, Netherlands) with Cu Kα radiation (*λ* = 0.1542 nm; 40 KV; 40 mA) at a scanning rate of a step size of 0.03° in the 2*θ* range from 5° to 50°.

Fourier transform infrared (FT-IR) spectra were conducted on a Nicolet-200SXV FT-IR machine (Nicolet, Waltham, MA) to identify the chemical structure of DA-M powder. Infrared spectra were recorded in a wave number range of 400–4000 cm^−1^.

### *In vitro* release behavior

To determine the release kinetics of DA from DA/MPEG-PCL nano-micelles, 500 μL of DA-M and free DA with a DA concentration of 0.5 mg/mL were placed in dialysis bags (molecular mass cutoff is 1.0 kDa and the dialysis area is 10 cm^2^), respectively. The dialysis bags were incubated in 30 mL of phosphate-buffered saline (PBS, pre-warmed to 37 °C, pH = 7.4) containing Tween-80 (0.5% wt) at 37 °C with gentle shaking (100 rpm). At pre-determined time points, all the release media were removed and replaced by pre-warmed fresh media. The released drugs were quantified using HPLC. All results were the mean of three samples, and all data were expressed as the mean ± SD.

### Surgical protocol

The rats were anesthetized with an intraperitoneal injection of chloral hydrate (10 wt%, 0.3 ml/100 g, Kelong, Chengdu, China). Immediately prior to the surgery, the animal’s back was shaved and aseptically prepared with povidone-iodine. The animals were placed on a heating pad to maintain body temperature at 37–38 °C. A multilevel laminectomy was performed at T9-10 thoracic vertebrae. Dorsal muscles were cut and maintained on the side using retractors. And then, a laminectomy at the T9-T10 vertebral levels was performed. The dura was incised longitudinally and pulled laterally with exposed dorsal surface of the spinal cord (Schematic figure). After all, a right spinal cord hemi-transection was realized by the use of a sharp blade. Finally, the muscle layers and skin were closed with suture after confirm that the surface of the tissue was free of meninges or blood clots.

Animals were assigned to the following treatment groups: (1) the sham groups (*n* = 7) in which a surgery was performed without injuring the spinal cord, (2) rats with hemisected spinal cord were randomly assigned to four groups (12 rats per group) that received the DA-M (6 mg/kg DA), blank micelles, free DA (6 mg/kg DA) and NS, respectively, by intravenous injection into the tail at the sixth hour and fourth day after surgery. Before intravenous injection, lyophilized powder was redissolved in normal saline with 1 mg/ml DA at 60 °C.

### Magnetic resonance imaging (MRI)

All experiments were performed on a 3.0 T clinical MR scanner (Siemens Trio Tim), using a rat phased array RF coil typed as CG-MUC18-H300-AS (Chengguang Medical Technologies Co., LTD, ShangHai, China). After general anesthesia, every rat was placed on the fixation system in the prone position to minimize the respiratory movements, meanwhile obtain a reproducible position in order to acquire the unique imagines. The spine MRIs were scheduled at 1 and 4 weeks post-SCI. Images of the spinal regions were acquired in the sagittal planes. The sequence protocol performed at each evaluation included *T1*- and *T2*-weighted, 256 × 256 matrix, field of view (FOV) 80, slice thickness 1 mm, TE 13/92 ms and TR 505/3000 ms, respectively.

### Basso Beattie Bresnahan (BBB)

Motor behavior was evaluated by using the Basso Beattie Bresnahan (BBB) score (Basso et al., 1995). The evaluation was performed using a double-blind method, and the average scores in each group were calculated. For each group, the BBB scores were evaluated at a day pre-operation and 1, 7, 14, 28, 56 and 84 days post-injury.

### Histopathologic examination

After BBB evaluation and MRI, rats in each group were sacrificed at 1 week, 2 weeks, 1 month and 3 months and transcardially perfused with 200 ml PBS, followed by 300 ml 4% paraformaldehyde in PBS. Spinal cords about 4 cm centered on the lesion were dissected and post-fixed in 4% paraformaldehyde overnight. Coronal sections (5 μm) were then stained with hematoxylin-eosin (H&E). Evaluation of damaged tissue was performed on coronal sections (50 μm apart) along the rostrocaudal axis. The areas were manually traced and quantified using ImageJ software (National Institutes of Health, Bethesda, MD). The lesion volume was obtained by the sum of total lesion area multiplied by distance between the sections.

Other series were immunostained with antibodies against glial fibrillary acidic protein (GFAP) (4466, CST) to recognize reactive astrocytes, Tuj-1 (5666, CST) antibody to detect axons, laminin antibody (L9393, Sigma) to investigate the formation of glial scar. All sections were analyzed using light microscopy (Olympus BX 45, Olympus, Hamburg Germany) by two pathologists in a blinded manner. Quantification of GFAP, laminin and Tuj-1 was performed by counting the number of positive cell in ten high-power visual fields random photos per section and five sections from each rat.

### Statistical analysis

The statistical analysis was carried out using SPSS 15.0 software (Chicago, IL). Comparisons of experimental data in different groups were performed using one-way analysis of variance (ANOVA), and results of *p* < 0.05 were considered statistically significant.

## Results

### Preparation of DA-M

**Figure 1.**
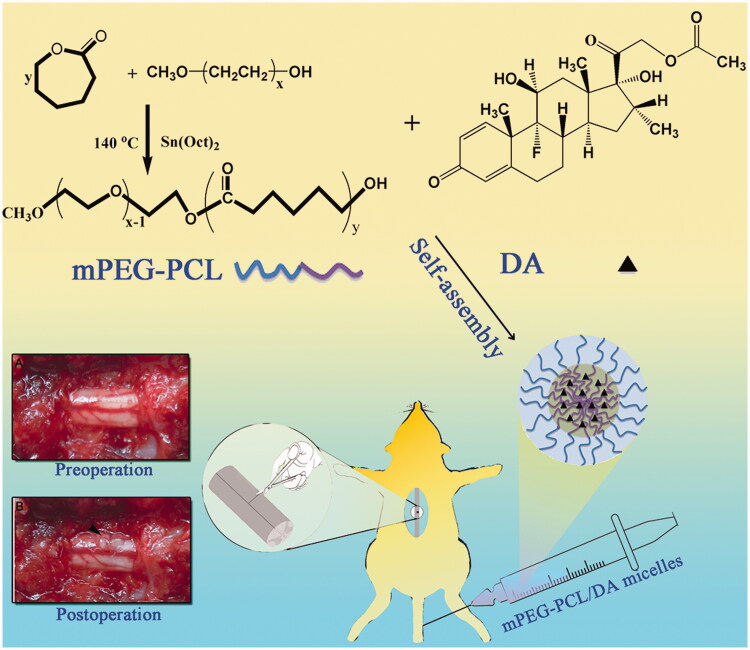
Schematic illustration of the synthesis scheme of MPEG-PCL, the formation of polymeric micelles from MPEG-PCL/DA, and schema of hemisection of rat SCI model (A: Preoperative; B: Postoperative).

**Figure 2.**
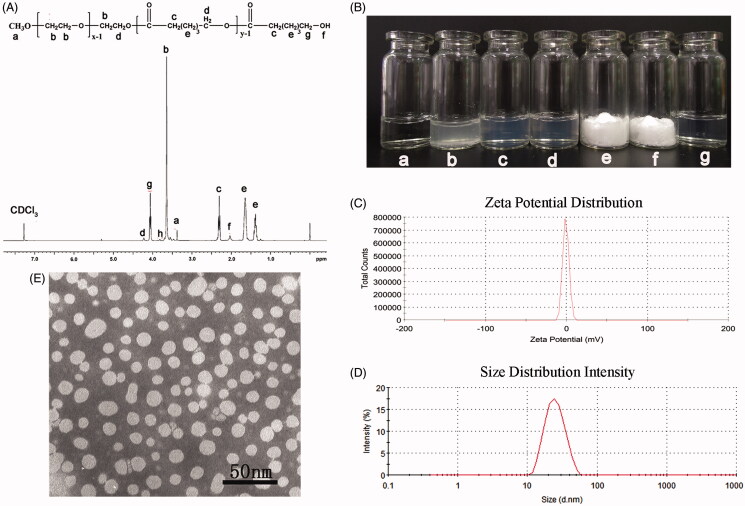
Preparation and characterization of DA-M. (A) Proton nuclear magnetic resonance spectra of MPEG-PCL; (B) Photographs of several solutions. (a) Water; (b) DA aqueous solution; (c) blank MPEG-PCL micelles at the concentration of 20 mg/ml (w/v); (d) DA-loaded micelles at the concentration of 20 mg/ml (w/v); (e) freeze-dried blank MPEG-PCL micelles; (f) freeze-dried DA-loaded micelles; (g) redissolved resolution of freeze-dried DA-loaded micelles at the concentration of 10 mg/ml (w/v); (C) zeta potential of DA-M; (D) particle size distribution of DA-M; (E) TEM image of DA-M.

After the successful synthesis of MPEG-PCL (Figure 1, Figure 2A), The DA/MPEG-PCL micelles were prepared by a self-assembly method. DA and MPEG-PCL copolymer were codissolved in dichloromethane/acetone (v/v = 1:4) and then were evaporated in rotary evaporator under gradually increasing vacuum degree. DA could distribute in MPEG-PCL copolymer (Figure 1) during the evaporation process. Subsequently, the evaporation was dissolved in normal saline to self-assemble into DA-M.

### Characterization of DA-M

The DA/MPEG-PCL micelles were characterized in detail. As shown in Figure 2(B), the solubility of DA in water (Figure 2B(b)) was poor, while the clear solution of blank micelles ((Figure 2B(c), 20 mg/mL) and DA micelles ((Figure 2B(d), 20 mg/mL) could be observed, indicating DA micelles gained good water solubility. Zeta potential of DA-M was 1.32 ± 0.4 mV (Figure 2C), which indicated that the surface charge could be regarded as neutral. And, the average particle size of obtained DA-M was 25.9 ± 2.7 nm, with polydispersity index (PDI) of 0.179 ± 0.024. According to the particle size distribution spectrum shown in Figure 2D, DA-M had a very narrow particle size distribution. Morphological analysis via TEM showed that the DA/MPEG-PCL particles, dissolved in aqueous solution, were spherical and had a mean diameter of approximately 25 nm (Figure 2E). The diameter of DA-M observed by TEM was in good agreement with the results of DLS. Microstructure of DA-M observed by TEM, as well as the particle size analysis, demonstrated that the prepared DA-M was stable and could be well-dispersed in aqueous solution. Furthermore, with the increasing DA content, a reasonable increase in drug loading was observed, leading to a decrease in encapsulation efficiency and stability time (Table 1), which might have been caused by the lack of a sufficient amount of dissolved MPEG-PCL to coat and stabilize the DA/MPEG-PCL micelles.

**Table 1.** Drug loading, encapsulation efficiency and stability time of micelles at various weight ratios.

DA/MPEG- PCL (W/W)	Drug loading (DL) (%)	Encapsulation (EE) (%)	Stability (*h*)
2:98	1.83 ± 0.03	91.43 ± 1.28	8
5:95	4.35 ± 0.06	87.99 ± 1.31	2
8:92	6.94 ± 0.03	86.60 ± 0.39	1.5
10:90	8.70 ± 0.26	85.74 ± 2.81	0.5
15:85	11.75 ± 0.11	75.43 ± 0.77	0.1

**Figure 3.**
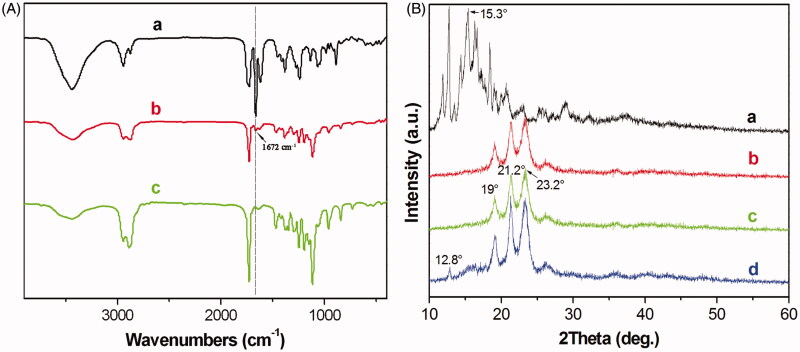
(A) FT-IR spectra of (a) DA, (b) freeze-dried of MPEG-PCL/DA micelles powder and (c) freeze-dried MPEG-PCL micelles powder; (B) X-Ray diffraction spectra of (a) DA; (b) MPEG-PCL; (c) freeze-dried MPEG-PCL/DA micelles powder; (d) DA mixture with MPEG-PCL powder.

Figure 3A shows the FT-IR spectra of pure DA (Figure 3A(a)), DA-M (Figure 3A(b)) and blank MPEG-PCL micelle (Figure 3A(c)). In the spectrum of the pure DA, the strong peak at 1672 cm^−1^ were assigned to the characteristic of C = C bands stretching vibrations, which could not be detected in the spectrum of the blank MPEG-PCL. However, in the spectrum of DA-M, the peak at 1672 cm^−1^ represents shift lower of C = C bands. The FT-IR spectroscopy shows that the main characteristic bands of DA and MPEG-PCL copolymer could be seen in the DA-M. There are no appreciable changes in the FT-IR spectra of the blends with respect to the addition of each component. Figure 3B shows a crystallographic assay performed by XRD. In comparison with the XRD diagrams of DA (Figure 3B(a)), blank MPEG-PCL copolymer (Figure 3B(b)), DA-M (Figure 3B(c)) and the physical mixture of DA and the MPEG-PCL copolymer (Figure 3B(d)), the absence of specific diffraction peaks in the XRD diagram of DA-M indicated that DA was completely, amorphously encapsulated within the core-shell structure of the particles.

### *In vitro* release behavior

**Figure 4.**
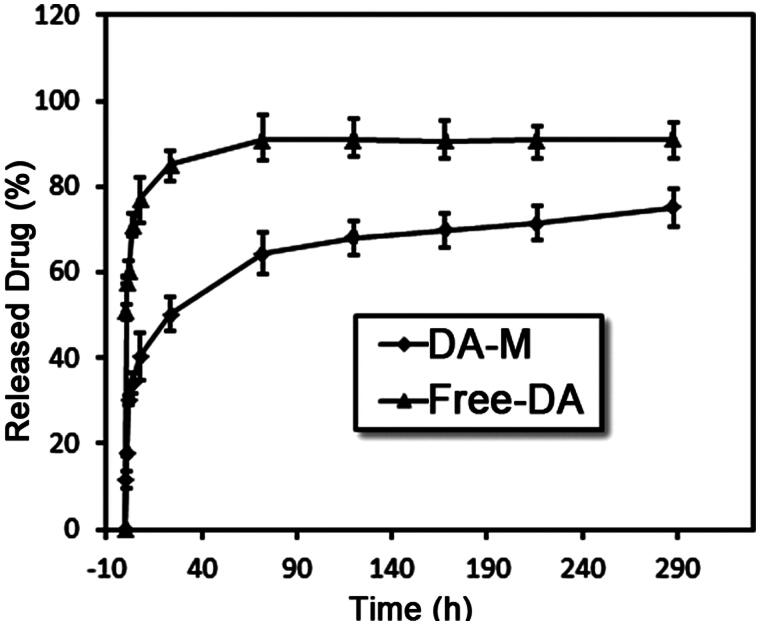
Drug release profiles of free DA and DA-loaded micelles in PBS solution at pH = 7.4.

As show in Figure 4, *in vitro* release behavior of DA and DA-M was investigated, and the results were significantly different. DA could be sustained release from DA-M, however, the rate of DA released from DA-M was much slower than free DA. As shown in Figure 4, 40.34 ± 4.94% of DA was released from DA-M in the first 8 h, which was significantly lower than that in free DA group (77.08 ± 5.59%, *p* < 0.05). Furthermore, at the end of this study, the cumulative release rate of DA micelles was 75.16 ± 3.99%, which was much lower than that of free DA (90.99 ± 4.45%, *p* < 0.05). This delay of drug release indicates potential applicability of the DA-M as *in situ* sustained drug-delivery system.

### Magnetic resonance imaging

**Figure 5.**
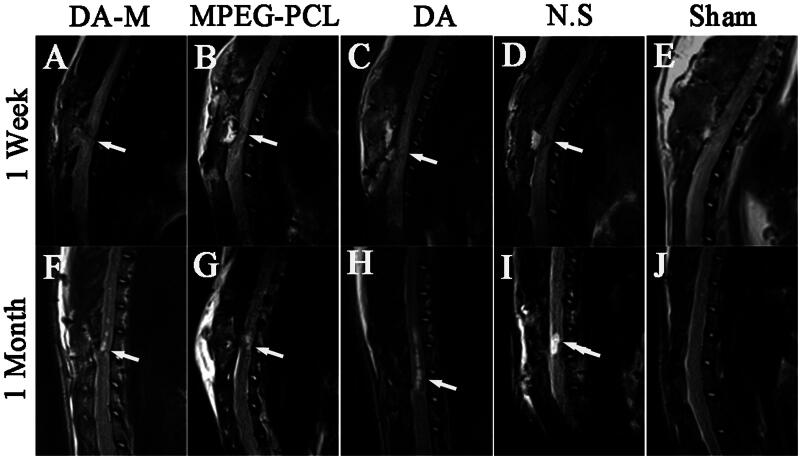
Magnetic resonance images of hemisection of rat SCI at 1 week and 1 month of post operation. White arrows indicate the site of injury.

To provide dynamically relevant evidence of the pathological change in SCI, a 3.0T MRI unit was applied for the experimental analysis of SCI, which is extensively used to assess patients with spinal cord disorder in the clinic, as shown in Figure 5. At 1 week after injury, MRI showed the injury site with hyperintensity surrounding it on *T2*-weighted images. Moreover, there were no significant differences among each group. Furthermore, the MRI obtained at 4 weeks after injury showed typical pathological changes and was characterized by a fluid-filled cyst with the highest intensity on *T2*-weighted images. However, the volume of cyst in DA-M group was significantly smaller than other SCI groups.

### BBB score

**Figure 6.**
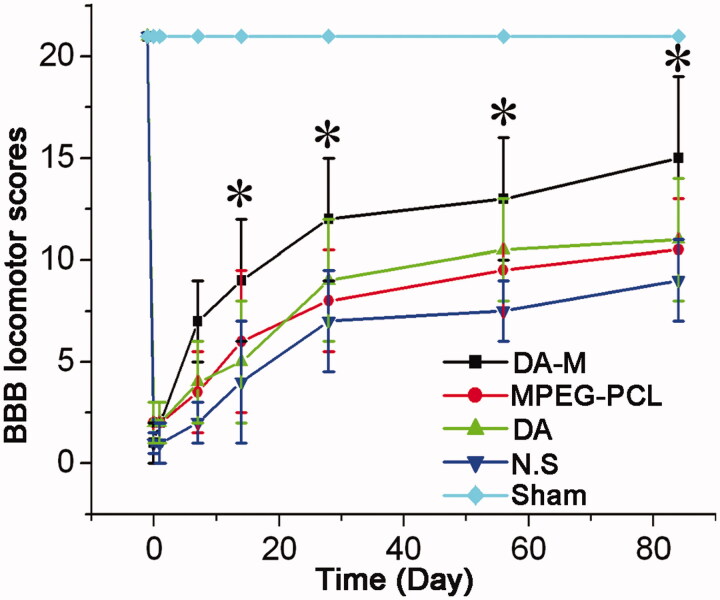
BBB locomotor scores of each group at 1 week, 2 weeks, 1month and 3 months. Significance was assessed compared to the other groups for each time point assayed by one-way analysis of variance. Quantitative data are represented as mean ± SD. *p* Values less than 0.05 were considered significant (*p < 0.05).

We evaluated motor function by means of serial BBB score analysis. The hind limbs of all experimental groups except sham group were paralysis after the operation. At one week post-SCI, the average BBB score was homogeneous between DA-M group, blank micelle group, free DA group and NS group (*p* > 0.05) (Figure 6). Notably, significant differences gradually emerged between each groups after 2 weeks post-SCI (*p < 0.05). During 3–12 weeks, the BBB score of hindlimb movement increased at every observation point, and the score of DA-M group still showed significantly higher value than the blank micelle group, free DA group and NS group.

### Histological observation

**Figure 7.**
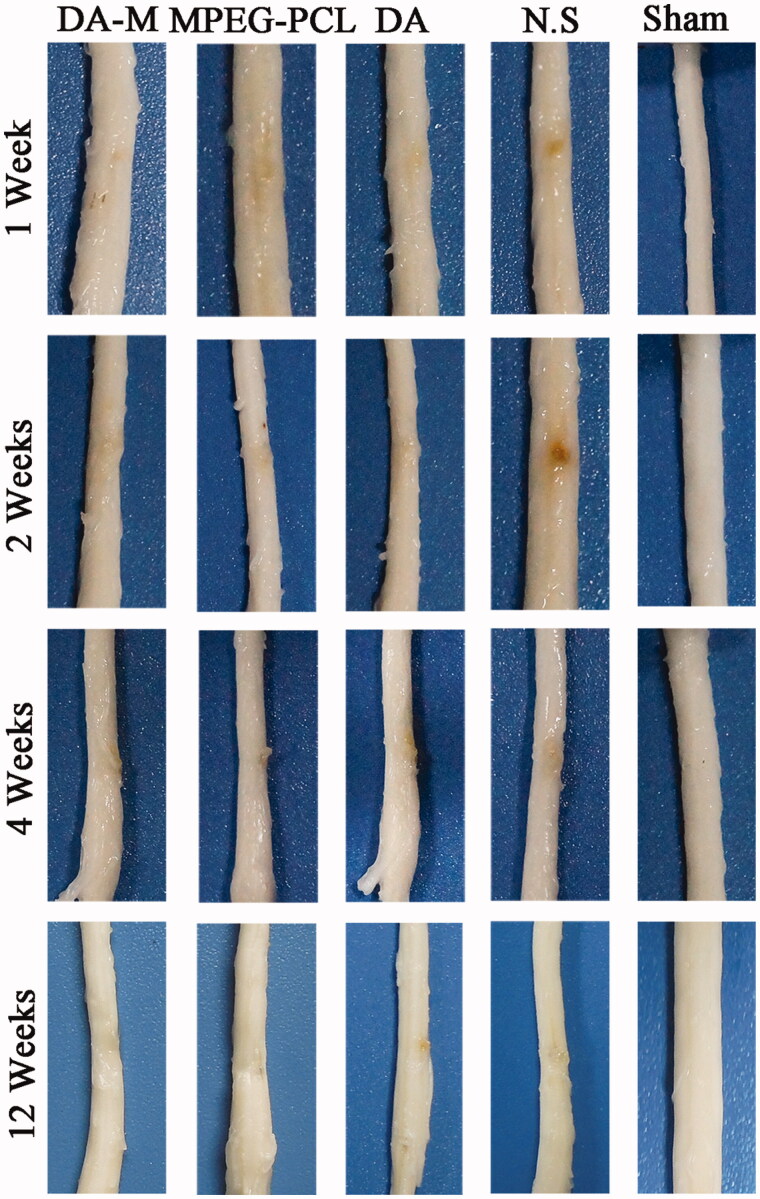
Representative photos of injured spinal cord in each group at 1 week, 2 weeks, 1month and 3 months.

**Figure 8.**
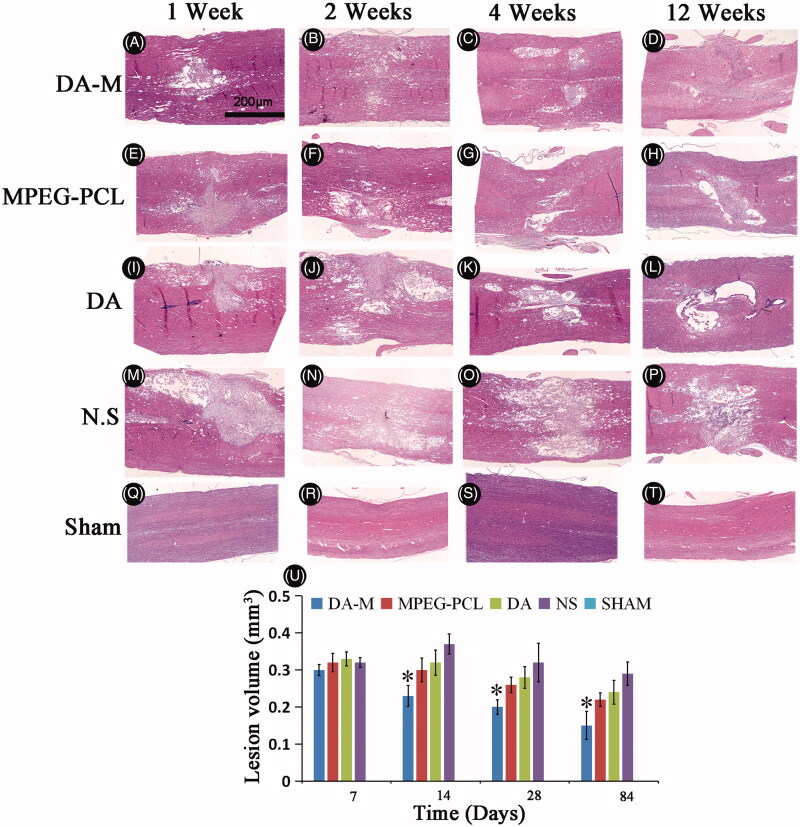
Photograph of injured spinal cord with hematoxylin-eosin stain in each group at 1 week, 2 weeks, 1month and 3 months (A–T). Quantitative comparison of the lesion volumes in DA-M-treated and other groups (U). Significance was assessed compared to the other groups for each time point assayed by one-way analysis of variance. Quantitative data are represented as mean ± SD. *p* Values less than 0.05 were considered significant (*p < 0.05). Scale bars represent 200 μm.

Using hemisection SCI model in rats, we further characterized the histopathological changes in injured spinal cords 1 week, 2 weeks, 4 weeks and 12 weeks post-operation. As showed in Figure 7, during the first two weeks, the acute injury stage, the injury site of spinal cord in each group show obviously hemorrhage and congestion. While at 4 and 12 weeks, the chronic injury stage, the congestion and swelling disappeared replaced by malacic foci and scar tissue. When investigated by H&E staining (Figure 8) and observed under microscope, nonlesion spinal cord was in the sham control group, which showed amounts of neuron and gliocyte, cells and axon arrange close together under normal condition. However, traumatic injury results in disorganized cells with extensive parenchymal hemorrhage, swelling and necrosis in injury site. Interestingly, administration of DA-M could significantly reduce the volume of the spinal cord lesion and cavity formation in injury site as compared to the other treated groups (Figure 8U).

**Figure 9:**
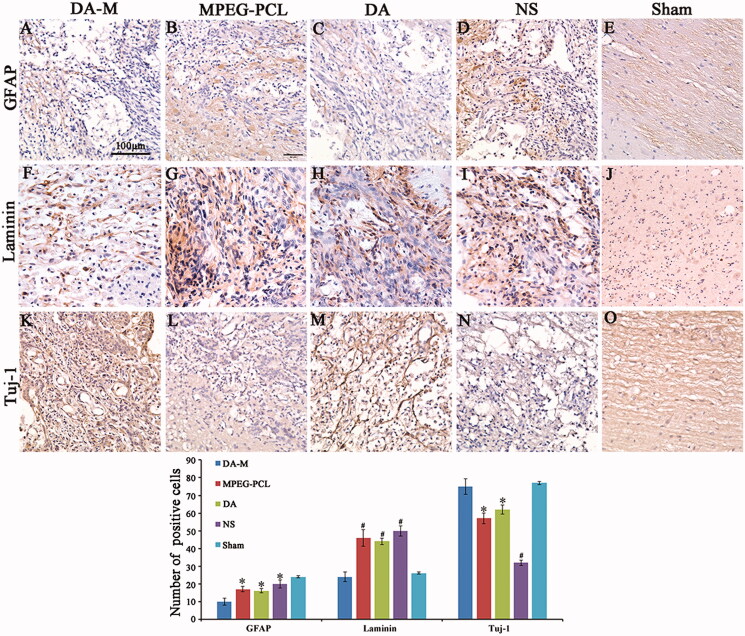
(A–E). GFAP, (F–J) Tuj-1 and (K–O) laminin immunohistochemical analysis. Spinal cord sections from the groups treated with intravenous DA-M, free DA, blank MPEG-PCL micelles and normal saline (NS) were subjected to immunohistochemistry to detect GFAP, Tuj-1 and laminin. (P) Quantification comparison of GFAP, laminin and Tuj-1positive cells in DA-M-treated and other groups. Significance was assessed by one-way analysis of variance. Quantitative data are represented as mean ± SD. *p* Values less than 0.05 were considered significant (*p < 0.05, #*p* < 0.01). Scale bars represent 100 μm.

To determine the anatomical basis of observed functional recovery, we examined several key parameters that were associated with tissue damage and repair by using immunohistochemical analysis (Figure 9). These parameters included densities of astrocytes, axons and formation of glial scar at 3 months (12 weeks) post injury. Astrocytes, which play a major role in the formation of gliosis after SCI, were visualized using glial fibrillary acidic protein (GFAP) antibodies (Figure 9A–E). The immunoreactivity of GFAP in the DA-M group was significantly less than that in the free-DM, blank-DM and NS group (*p* < 0.05, Figure 9P). Scar tissue in the injury site of spinal cord poses a key impediment to regenerating axons. We further investigate the formation of glial scar by using laminin (Figure 9F–J). The results disclosed that rats after SCI treated by DA-M show significant reduction of laminin positive fibrotic scar tissue in injury site (*p* < 0.01, Figure 9P). To determine whether the reduced immunoreactivity of astrocytes and glial scar benefit the survival of axons, we quantified their densities using Tuj-1 immunofluorescence. The results presented in Figure 9(K–O) show that DA-M treatment increased the number of spared axons in the epicenter than that in the free-DM (*p* < 0.05), blank-DM (*p* < 0.05) and NS group (*p* < 0.01). These results collectively show that DA-M treatment not only suppressed the astrogliosis and glial scar formation, but also protected the axons.

## Discussion

Dexamethasone acetate (DA) is a synthesized glucocorticoid, which has been used as a neuroprotective drug after SCI for a long time (Kiwerski, 1993; Kwiecien et al., 2016). Kiwerski had reported that the patients had been given dexamethasone over 24 h were reassigned to a better neurologic outcome when treated with dexamethasone compared with controls (Kiwerski, 1993). However, as the same as other glucocorticoid, DA has poor water solubility and is almost insoluble in most physiologically compatible and pharmaceutically acceptable solvents, both of which limit its clinical applications (Chopra et al., 2012). In addition, the BSCB protects and regulates the parenchyma and provides a specialized microenvironment for the cellular constituents of the spinal cord (Bartanusz et al., 2011). In order for drugs such as DA, or other glucocorticoids, to reach therapeutic levels at the injury site, an extremely high systemic dose is required (Bracken et al., 1997). These high doses can result in toxicity and systemic side effects (Suberviola et al., 2008). Furthermore, intravenous administration of free drugs also faces challenges from renal clearance of drug, limited drug circulation time and drug degradation (Allen & Cullis, 2004; Fan et al., 2015; Gao et al., 2015). Therefore, increasing treatment efficiency of DA in company with decrease in its side effect is an important research goal that could facilitate its clinical use.

Fortunately, MPEG-PCL micelles are able to solve the problem of DA’s poor water solubility and enhance its treatment efficiency (Duan et al., 2015; Wang et al., 2016). MPEG-PCL is an amphiphilic block copolymer composed of hydrophilic and hydrophobic segments, which has a tendency to self-assemble into micelles in a specific solvent (Gao et al., 2015; Li et al., 2016). Moreover, the hydrophobic blocks can serve as a container for DA in the central, the hydrophilic blocks form a shell contacting with blood components or aqueous solvent. In previous studies, with the advantage of biodegradability, biocompatibility and lower toxicity MPEG-PCL has been widely employed as hydrophobic drug-delivery system such as deliver doxorubicin, curcumin and quercetin for cancer therapy (Wang et al., 2015; Ehlerding et al., 2016; Zheng et al., 2016). Moreover, the results have been disclosed that MPEG-PCL could enhance the anti-cancer effect of these drugs, indicating that it was a good drug-delivery system (Chen et al., 2016; Cheng et al., 2016; Rezazadeh et al., 2016). In this study, we innovatively encapsulated DA into MPEG-PCL micelles, and found it could play a better role in neuroprotection of SCI.

Additionally, MPEG-PCL could work as membrane sealing agents, which could synergy with dexamethasone neuroprotective effects together. During the primary injury of SCI, the acute mechanical damage to the spinal cord breaks neuronal membranes and causes Ca^2+ ^influx into cells (Snyder & Teng, 2012; Tyler et al., 2013). The latter readily triggers a series of adverse events compromising the plasma membrane’s essential role, progressive disruption of nearby neuronal tissues, to eventual disruption-induced cell and tissue necrosis (Shi et al., 2010; Snyder & Teng, 2012; Tyler et al., 2013). In the previous study, it has been approved that PEG could work as membrane-sealing agents which has been used to restore compound action potential (CAP) in *ex vivo* tissues and recover behavioral functions *in vivo* (Borgens & Bohnert, 2001). However, due to the viscosity of high molecular weight (MW) and the toxicity of low MW after the degradation of PEG, its administration must be limited in concentration and in timing after acute clinical neurotraum (Brent, 2001). Thus, shi et al presented a new approach that allows effective membrane repair and functional recovery in SCI animals (Shi et al., 2010). Instead of using individual polymers, the author chose PEG–polyester micelles, which are spherical assemblies of di-block copolymers containing a hydrophilic PEG shell and a hydrophobic inner core. Intravenously injected PEG–polyester micelles effectively recovered locomotor function and reduced the volume and inflammatory response of the lesion in injured rats, without any adverse effects (Borgens & Bohnert, 2001).

In order to maximize the role of DA-M in treatment of SCI, excellent characteristics in suspension are needed. Owing to the encapsulation of DA in MPEG-PCL micelles, the DA concentration in aqueous solvent eventually significantly elevated compare with free DA. It is known that micelles tend to form aggregates due to their high surface/volume ratio. In addition, a stable suspension of micelles can be influenced by electrostatic interactions and/or steric hindrance. It is considerable to find out that the surface charge of DA-M was nearly neutral, thus a stereospecific blockade should contribute to the stability of DA micelles. In addition, the great difference in hydrophobicity between the hydrophobic PCL segment and hydrophilic PEG segment allows the formation of micelles with a PCL core and a PEG shell. The PEG segments are located at the surface of the micelles and provide limited affinity among the particles, preventing the formation of aggregates (Gao et al., 2015). We further find out that MPEG-PCL encapsulating DA showed small particle size, good homogeneity, high EE and high DL, as well as lasting *in vitro* release behavior.

To further study the characteristics of DA-M, FT-IR and XRD were performed. The FT-IR spectroscopy shows that the main characteristic bands of DA and MPEG-PCL copolymer could be seen in the DA-M. There are no appreciable changes in the FT-IR spectra of the blends, which represents those two compounds form completely immiscible blends. The results of XRD indicate that DA was completely, amorphously encapsulated in the core-shell structure of the MPEG-PCL micelles, and was not influenced by the crystallization of MPEG-PCL.

To determine whether DA-M could improve functional outcomes in live animals, we performed a behavioral study using SD rats with hemisection-injured spinal cords and evaluated the hindlimb functional recovery using the Basso Beattie Bresnahan locomotor rating scale. The results revealed that DA-M group show significantly better hindlimb functional recovery than other treatment group. We suspected that DA-M with nanoscale particle size could prevent glomerular filtration, which further extends the retention of micelles in the blood. Eventually, the carried drug DA of DA-M could act as a better drug with neuroprotective effect. Furthermore, through magnetic resonance scan analysis, we found that smaller cyst was formed after treated by DA-M, which play an important role in hindlimb functional recovery.

We further examined whether intravital micelle treatment would result in tissue protection. By histological investigation of sliced spinal tissues, DA-M could significantly reduce the volume of the spinal cord lesion and cavity formation in injury site as compared to the other treated groups. Moreover, at week 12 after hemisection injury, DA-M make the injury site of spinal cord as more Tuj-1 positive cell, which shows better neuroprotective effect. Meanwhile, less amount of GFAP and laminin positive cell illustrates that DA-M has strong inhibitory effect of glial scar. We suspected that the excellent tissue protection might attribute to their nanoscale size and flexibility, which could easily penetrate into the injury site while staying and releasing DA slowly to treat the injured spinal cord.

## Conclusions

In this work, we reported the preparation of biodegradable polymeric micelles entrapping DA and their neuroprotective effect *in vivo*. Compared to free DA, DA micelles of small particle size and good homogeneity demonstrated lasting *in vitro* release behavior. In the hemisection SCI model, DA micelles were more effective in promoting hindlimb functional recover, reducing glial scar and cyst formation in injury site, decreasing neuron lose and promoting axon regeneration. Therefore, polymeric micelles encapsulating DA with enhanced neuroprotection may become a brilliant aqueous formulation for intravenous application of future SCI therapy.
